# The Effect of Nutrition and Exercise on Body Composition, Exercise Capacity, and Physical Functioning in Advanced CKD Patients

**DOI:** 10.3390/nu14102129

**Published:** 2022-05-20

**Authors:** Maryam Ekramzadeh, Domenico Santoro, Joel D. Kopple

**Affiliations:** 1 Nutrition Research Center, Department of Clinical Nutrition, School of Nutrition and Food Sciences, Shiraz University of Medical Sciences, Shiraz 71348-14336, Iran; maryam.ekramzadeh@lundquist.org; 2 Division of Nephrology and Hypertension, Lundquist Institute, Harbor-UCLA Medical Center, Torrance, CA 90502, USA; 3 Department of Clinical and Experimental Medicine, Nephrology and Dialysis, University of Messina, 98100 Messina, Italy; domenico.santoro@unime.it; 4 David Geffen School of Medicine, University of California, Los Angeles UCLA, Los Angeles, CA 90095, USA; 5 Fielding School of Public Health, University of California, Los Angeles UCLA, Los Angeles, CA 90095, USA

**Keywords:** chronic kidney disease, protein energy wasting, malnutrition, nutritional supplements, physical activity, hemodialysis, physical performance, body composition, muscle mass

## Abstract

Patients with stages 4 and 5 chronic kidney disease (CKD), and particularly chronic dialysis patients, commonly are found to have substantially reduced daily physical activity in comparison to age- and sex-matched normal adults. This reduction in physical activity is associated with a major decrease in physical exercise capacity and physical performance. The CKD patients are often physically deconditioned, and protein energy wasting (PEW) and frailty are commonly present. These disorders are of major concern because physical dysfunction, muscle atrophy, and reduced muscle strength are associated with poor quality of life and increased morbidity and mortality in CKD and chronic dialysis patients. Many randomized controlled clinical trials indicate that when CKD and chronic dialysis are provided nutritional supplements or undergo exercise training their skeletal muscle mass and exercise capacity often increase. It is not known whether the rise in skeletal muscle mass and exercise capacity associated with nutritional support or exercise training will reduce morbidity or mortality rates. A limitation of these clinical trials is that the sample sizes of the different treatment groups were small. The aim of this review is to discuss the effects of nutrition and exercise on body composition, exercise capacity, and physical functioning in advanced CKD patients.

## 1. Introduction

Patients with advanced chronic kidney disease (CKD), and especially those undergoing chronic dialysis, display decreased physical activity [1]. Reduced activity is considered to have many causes including superimposed physical illness, psychological stress [2,3], protein energy wasting (PEW), frailty [4], and inflammation [5]. Reduced physical activity and impaired physical functioning may affect the quality of life, morbidity, and mortality in this population [6,7]. Evidence shows that rehabilitation interventions to increase exercise capacity in CKD patients may reduce the risk of morbidity and mortality [8]. Many studies have been conducted regarding the effect of exercise training or nutritional supplementation on physical functioning, physical activity, and nutritional indices [9,10,11,12,13]. In general, these studies show that nutritional supplements or exercise training may enhance nutritional intake and improve muscle mass and exercise capacity in advanced CKD patients. The aim of this review is to describe the effects of nutrition and exercise on body composition, exercise capacity, and physical functioning in advanced CKD Patients.

## 2. Methods

The databases PubMed, Cochrane, Science Direct, and Google Scholar were searched using the following keywords: physical activity, muscle strength, muscle mass, physical performance/function, body composition, exercise capacity, PEW, frailty, exercise, endurance (cardiorespiratory) and resistance training, and nutritional supplementation. These words were often searched for alone or in combination with the terms CKD, end-stage renal disease (ESRD), and/or dialysis. More than 400 articles were identified. The search for clinical studies was limited to randomized trials in ESRD patients undergoing chronic dialysis (hemodialysis or peritoneal dialysis, HD or PD) between 2005 and 2021. Twelve randomized, controlled clinical trials of nutritional support with or without exercise training were published between 2013 and 2021. These are listed in Table 1. Between 2005 and 2020, 17 randomized trials and one nonrandomized trial evaluated exercise training alone. These manuscripts are referred to in Table 2. In both tables, the outcomes evaluated were body composition parameters, nutritional indices, exercise capacity, and physical function. Studies that used different outcome measures than these were excluded from this analysis.

## 3. Physical Inactivity and Physical Function in CKD Patients

People with advanced chronic kidney disease (CKD) tend to be more physically inactive, have a sedentary lifestyle [1,39], and display impaired physical exercise capacity [40,41] and physical performance [40,42]. The degree of inactivity and impaired performance is heavily dependent on the severity of CKD [1,43,44,45]. In general, people with CKD stages 1, 2, and often 3a (Glomerular Filtration Rate (GFR) < 60 to ≥45 mL/min/1.73 m^2^) who do not have much proteinuria and who do not have other symptomatic, debilitating comorbidities (e.g., no heart or lung failure, active cancer, vasculitis, obesity, vascular insufficiency, or uncontrolled hypertension) may have an exercise capacity which is normal or, if the person chronically exercises, above normal [46,47]. By contrast people with CKD stage 3b, and particularly CKD stages 4 and 5 and chronic dialysis patients, which we will refer to in this paper as advanced CKD, commonly lead more physically inactive lives [43,46,47,48,49]. In general, the more severely reduced the glomerular filtration rate (GFR), the more physically inactive [1,43,50] and the more severely impaired the exercise capacity of many CKD patients [37,46]. These matters are considered to be of major clinical importance because physical dysfunction, muscle atrophy, and reduced muscle strength are all associated with poor quality of life and increased morbidity and mortality in CKD and ESRD patients [4,6,51,52,53,54,55]. 

First, to define some terms: Physical activity (PA) is defined as any bodily movement that is produced by the contraction of skeletal muscle and that increases energy expenditure above basal levels [53]. Physical functioning (PF) is the ability to perform the normal activities of daily living and is generally assessed by simple tests of physical activity [7]. It characterizes an essential component of health status and depends on the sensory and motor skills necessary for usual daily activities [56]. PF may be assessed by the usual gait speed test, the handgrip strength, the timed up-and-go test, and the 6-min walk test [55]. Physical performance (PP) is one’s ability to carry out specific physical tasks that might be necessary in the course of normal daily living (e.g., walking and stair-climbing) and is generally assessed by the ability to perform these activities [56]. Exercise capacity is the maximum level of oxygen consumption achieved during maximum exercise [56]. Exercise capacity is often expressed as metabolic equivalents (METs) which is defined as the multiple of a person’s basal oxygen consumption at rest. Individuals consume a basal amount of oxygen in the performance of their resting metabolism. This basal oxygen consumption is generally measured in the morning after an overnight fast (i.e., in the post-absorptive state) while lying comfortably in bed. This level of oxygen consumption is referred to as one metabolic equivalent or one MET. One MET equals the resting metabolic rate of a person. One MET will vary somewhat from person to person, and is approximately 3.5 mL O_2_/kg/min [56].

Another measure of exercise capacity is called maximal oxygen consumption (VO_2max_) which is the highest oxygen consumption observed during graded exercise to physical exhaustion. Peak oxygen consumption (V_o2peak_) refers to a person’s peak oxygen consumption that is attained during a specific maximal exercise test and is more commonly used than VO_2max_ [56].

Advanced CKD patients are clearly less physically active than matched normal people [1,47,57]. Although the World Health Organization recommends that adults exercise each week for at least 150 min of moderate intensity PA or 75 min of vigorous intensity PA or some combination therein, it is clear that the great majority of advanced CKD patients do not exercise at anywhere near this level [1,47,57,58]. The National Health and Nutrition Examination Survey (NHANES) III indicated that 28% of subjects with CKD (defined as eGFR < 60 mL/min/1.73 m^2^, stages 3–5), reported being physically inactive compared to 13.5% of the non-CKD population age range, sex, and race [59]. Using more objective methods of measuring physical activity, such as with accelerometers, Beddhu et al. found that patients with CKD (mean eGFR, 48.5 ± SD12.9 mL/min/1.73 m^2^) were sedentary more than two-thirds of the waking day compared to one-half of the waking day in individuals without CKD [60]. Stack et al. reported that 75% of new onset dialysis patients showed severe limitations in their ability to conduct vigorous activities, and 42% of these patients described severe limitations in moderate activities (e.g., moving a table or vacuuming) [61]. 

The United States Renal Data System Comprehensive Dialysis Study involving 1547 ambulatory patients new to dialysis indicated that physical activity levels were below the fifth percentile of healthy subjects matched by age and sex [62]. Physical performance tests related to activities of daily living that were conducted in 32 CKD patients (eGFR, 29.9 ± SD 17.0 mL/min/1.73 m^2^) showed that the mean maximal gait speed, and sit-to-stand performance tests, were lower, 85% and 79%, respectively, than normal sedentary reference values [63]. The findings of a Swedish study in 55 CKD individuals with a measured GFR ≤ 20 mL/min/1.73 m^2^ revealed that mean peak values of handgrip strength were 78% and 84% of the values of normal age-matched males and females, respectively [64]. In this same study, for the patients with eGFR ≤ 12 mL/min/1.73 m^2^, for every 1 mL/min/1.73 m^2^ further decrease in GFR, there was an increase in the proportion of patients who were not able to rise from a chair without the use of his/her arms [64]. Kim et al. found that in 72 hemodialysis (HD) patients, daily physical activity (assessed by an accelerometer over a 7 day period) and physical performance (measured by a 6-min walk test (6MWT), sit-to- stand test, and stair-climbing test) were about 60–70% of normal age- and sex-matched values [47]. 

The international DOPPS study (Dialysis Outcomes and Practice Patterns Study) reported that among 20,920 HD patients from 12 countries more than the half of the patients engaged in some type of exercise either less than 1 day per week or never [65]. Another study in 78 HD patients in Spain in which daily physical activity was measured for 6 consecutive days with a pedometer, indicated that 71% of HD patients were considered sedentary (<5000 steps/day) [66]. It might be argued that taking 4999 steps per day is not very sedentary. These patients actually took 3767 ± SD 3370 steps per day on non-HD days and 2274 ± SD 2048 steps per day on HD days [66]. In addition, 134 HD patients from five hemodialysis centers in France, Switzerland, Brazil, or Sweden were assessed by an armband activity monitor for 5 days for the number of steps they took each day [67]. These patients took an average of 5544 steps per day on non-HD days and 4620 steps per day on HD days [67].

Low physical functioning in advanced CKD patients is associated with reduced exercise capacity, especially at the time that they begin chronic dialysis therapy or after they are established on this treatment [37,46]. Reduced exercise capacity, lower peak oxygen consumption (VO_2 peak_), and impaired maximal oxygen consumption (VO_2max_) are very prevalent and often quite severe in CKD stages 3–5 and in chronic dialysis patients [40,41,46]. Moreover, in a national registry of nursing home residents whose functional status was monitored serially starting shortly after they commenced HD, a sharp and significant reduction in functional status of HD patients was observed by 12 months after the initiation of chronic dialysis [40]. As indicated above, one reason for such reduced physical function in advanced CKD patients may be that these individuals do not undertake much physical activity [47,68].

The degree of physical inactivity and deconditioning of peritoneal dialysis (PD) patients is similar to that of HD patients [1]. A few small studies in PD patients showed that VO_2 peak_ levels are similar to those of HD patients. Compared to the general population, PD patients also showed reduced physical function and lower physical activity levels [69]. Sixty-four automated peritoneal dialysis (APD) and continuous ambulatory peritoneal dialysis (CAPD) patients walked 4839 ± SD 3313 steps per day and were found to have sedentary behavior as defined by <5000 steps per day as measured by pedometers [66].

## 4. Frailty, Protein Energy Wasting and Chronic Kidney Disease

Perhaps no discussion of exercise capacity and physical function in CKD patients would be complete without a discussion of frailty in CKD [4,70,71]. Previously, the concept of frailty was generally applied to older adults and could be defined as, “(older adults or aged) individuals who are lacking in general strength and are unusually susceptible to disease or to other infirmity” [72,73]. Now frailty is used to characterize people of all ages who are debilitated and who often have other illnesses (e.g., CKD) [74]. The diagnosis of frailty has elicited much interest because it is associated with adverse clinical outcomes, morbidity, and mortality [4,72,75] and because it is potentially treatable [4,73,74].

Many criteria have been developed for diagnosing frailty and measuring its severity. This is especially the case for people with specific clinical disorders or disease states [72,74,76,77,78,79,80]. One set of criteria for diagnosing frailty in CKD patients, which was developed by Fried et al. and appears to be particularly useful, is the presence of at least three of the following five conditions: (1) unintentional weight loss, (2) self-reported exhaustion, (3) measured weakness, (4) low walking speed, and (5) low physical activity [63]. Using these criteria, frailty in CKD patients is associated with a substantially worse clinical prognosis [4,74,81,82,83]. The above five individual elements involved in the diagnosis of frailty, as well as the diagnosis of frailty itself, increase in prevalence as the GFR decreases [83,84]. Moreover, frailty is more prevalent in chronic dialysis patients [70,74,85]. 

It is noteworthy that both frailty and protein energy wasting (PEW) are common in advanced CKD and are both characterized by unintentional weight loss, physical debility, and low physical activity [4,86]. PEW is often present in CKD patients who have frailty and impaired exercise capacity [4]. PEW is defined as the loss of somatic and circulating body protein and energy reserves [87]. The term PEW is used rather than protein energy malnutrition because some causes of PEW are unrelated to inadequate nutrient intake. There is no universally accepted criteria for diagnosis of PEW [86,88]. One commonly proposed diagnostic criteria for PEW is the presence of at least one measure from at least three of the following four categories of protein–energy status: decreased serum concentrations of certain compounds, primarily such proteins as albumin or pre-albumin (transthyretin), low or decreasing body mass, low or decreasing skeletal muscle mass, or unintentional decrease in dietary protein or energy intake [89].

## 5. Causes of Decreased Physical Activity in CKD Patients 

The focus of this paper does not allow an extensive discussion of the causes or diagnosis of frailty or PEW in CKD patients. This subject has been extensively reviewed in other publications [4,86,87,88,89]. It should be emphasized that many of the causes of frailty and PEW are similar or identical, and these two syndromes often occur together in CKD patients [4].

Decreased daily physical activity, reduced exercise capacity, impaired physical performance, and frailty observed in advanced CKD patients can be the result of many potential interrelated factors that occur in advanced CKD. These may include the uremic syndrome per se, protein energy wasting [87], inflammation [5], anxiety, depression [2,3,46,47,90,91], hyperparathyroidism [92], bone disorders [49], metabolic acidosis and accompanying comorbidities that are often associated with CKD (e.g., diabetes mellitus, vasculitis, obesity, or heart failure) [4,47,68], anemia [93], HD-related fatigue, and time spent on dialysis [94]. A uremic myopathy [95,96] and hormonal derangements (i.e., decreased serum testosterone levels [97] or resistance to the actions of growth hormone [98,99] or insulin-like growth factor-I (IGF-I) [100,101], hyperglucagonemia [102,103], and hypothyroidism) [104,105] may also contribute to these disorders.

## 6. Results

### Nutrition and Exercise Training 

Many studies have examined the effects of nutritional support on skeletal muscle [15] or total body protein [15,106,107,108,109,110,111,112,113] synthesis in people with CKD. Most of these studies were conducted in patients undergoing HD rather than nondialyzed CKD patients or PD patients. Several reports indicate that nutritional supplements that provide protein or amino acids and energy may acutely increase skeletal muscle [15] or total body protein synthesis [15,106,107,108,109,110,111,112,113] in CKD patients. Increased protein synthesis has also been noted in HD patients when they undergo HD while receiving nutrients intravenously [108,109] or orally [15,106,107,110] as compared to when they are fasted.

The long-term effects of nutritional support are more likely to be manifested by changes in body composition than by increased measured rates of protein synthesis or degradation. Table 1 summarizes clinical trials of the effects on body composition in CKD patients who received nutritional support. Patients in these trials were more likely to have advanced CKD and were almost always chronic dialysis patients. In some but not all studies, the patients had protein energy wasting. In some trials conducted in HD patients, supplements were given only during HD treatment, whereas in other trials HD patients were offered the supplements daily. Furthermore, cardiopulmonary (aerobic) or resistance exercise training was offered in addition to nutritional supplements in several trials. In one trial, calorie restriction and aerobic exercise training were offered to stage 3–4 CKD patients to reduce their body fat [16]. In most, but not all trials, patients were randomized to one or more treatment protocols or to control groups. The treatment protocols in these clinical trials were usually of only a few weeks’ to several months’ duration.

The outcomes of these clinical trials were variable (Table 1). In a number of the studies, nutritional support with or without exercise was associated with a reduction in the number of patients who displayed evidence for malnutrition or inflammation. For example, the number of patients who displayed an abnormal malnutrition inflammation score (MIS) or who had evidence for PEW often decreased with nutritional support. Most of the studies indicated that nutritional support by itself or with some form of exercise training is often associated with an increase in muscle or fat mass or serum proteins, including serum albumin and pre-albumin (transthyretin). Sometimes serum proinflammatory cytokines decreased. However, usually there was no statistically significant difference between the patients who received nutritional support alone and those who underwent both nutritional support and some form of exercise training. Nutritional support with or without exercise training was not always associated with increased protein or fat mass.

Table 2 summarizes the results of randomized controlled trials of exercise training without supplemental nutrition. Again, most of these studies were conducted in HD patients. With exercise training, muscle mass in the exercising extremities often increased, especially if the exercise involved resistance training rather than cardiopulmonary exercise. Fat mass sometimes decreased or rose. Exercise capacity and physical function often improved with exercise training, and the magnitude of improvement often was impressive. Serum pro-inflammatory cytokine levels sometimes decreased. Again, it is possible that a greater increase in muscle mass and exercise capacity, and more reduction in body fat might have been demonstrated if the sample sizes of the patients studied had been greater or if the number of weeks or months of exercise training had been extended.

## 7. Discussion

The results of the studies presented in this manuscript indicate that nutritional supplements, exercise training, and the combination of the two treatments were often associated with improvement in nutritional (protein–energy) status or physical function in advanced CKD patients and especially chronic dialysis patients. However, in a number of the trials the improvement, although often statistically significant compared to the baseline assessment of the patients, was not significantly different from the changes in the controls.

One reason for the discrepancy in the results of these trials could be related to the protein–energy status and comorbidity of the patients. It would seem likely that CKD patients who had PEW, especially if the PEW was due to malnutrition, would be more likely to respond to nutritional therapy. The small sample sizes in these studies may be another reason that there are not more significant differences in changes in body composition between the nutritional support and control groups. The baseline inflammatory status and comorbidity of the patient groups often were not well defined, which may have prevented a statistically significant response to nutritional support and/or exercise training from being observed. This is especially the case because the sample sizes of the studies were small. Moreover, the composition of the nutritional supplements offered to these patients might not have been optimal. Various clinical factors such as acidosis, the characteristics of the dialysis procedures, inflammation, diabetes, and other comorbidities may also have influenced the response to nutritional supplementation, exercise training, or the combination of the two treatments [86,114,115].

Protein energy wasting, frailty, and impaired exercise capacity are each associated with increased morbidity and mortality in CKD patients [4,55,68]. An open question that was not addressed by these clinical trials is whether treatment to improve these three disorders will reduce morbidity and mortality in CKD patients (Table 1 and Table 2). Clinical trials with much larger sample sizes and much longer durations of treatments would be necessary to examine these questions. For logistical reasons, these types of trials will be harder to organize and fund.

## 8. Conclusions

Published data indicate that patients with stages 4 and 5 CKD, and particularly chronic dialysis patients, often have decreased exercise capacity and physical performance. Their daily physical activity is often substantially reduced in comparison to age- and sex-matched normal adults. Protein energy wasting and frailty are common in these patients. Many randomized controlled clinical trials indicate that when these individuals are treated with nutritional supplements, exercise training, or both procedures, their skeletal muscle mass and exercise capacity often increase. Further research is needed to determine whether exercise training and nutritional supplements or a combination of these two treatments will improve whole body protein or skeletal muscle mass, physical function, and exercise capacity. If muscle mass does increase with these treatments, it will become important to assess whether these treatments increase protein synthesis, reduce protein degradation, or do both. Ultimately, the value of nutrition supplementation and exercise training will be assessed by their effects, if any, on the quality of life, morbidity, and mortality rate of advanced CKD patients.

## Figures and Tables

**Table 1:** The effects of nutritional support with or without exercise training on body composition, exercise capacity, physical performance, and proinflammatory cytokines in advanced CKD patients.

Column	Reference, Study Design	No. of Patients	Type of Patients	Methods of Intervention	Duration	Significant Results
1	Limwanata et al., 2021 [13], randomized controlled trial	Rx1-26 Rx2-30 Control-24	Malnourished HD, age range 18–75 years	Rx1: ONS (370 kcal/day, 17 g protein, 42.2 g carbohydrate, 16.4 g lipid) Rx2: ONS (370 kcal/day, 16.6 g protein, 33 g carbohydrate, 19.8 g lipid) Control: dietary counseling	1 month	Significant changes within each group: Rx1: ↑ dietary intake of energy, protein, fat, fiber, and magnesium, ↑ mid-arm circumference, ↑ body weight, ↑ BMI, ↑ pre-albumin and albumin; ↓ MIS; no change in muscle mass, handgrip strength, percentage body fat, and triceps skinfold thickness Rx2: ↑ dietary intake of energy, protein, fat, fiber, and magnesium, ↑ mid-arm circumference, ↑ handgrip strength, ↑ body weight, ↑ BMI; ↓ MIS; no change in muscle mass, percentage body fat, triceps skinfold thickness, and pre-albumin and albumin Control: no change in any markers Two Rx groups vs. control: ↑ dietary intake of energy, protein, fat, fiber, and magnesium, ↑ albumin; ↓ MIS; no change in muscle mass, mid-arm circumference, handgrip strength, body weight, BMI, percentage body fat, triceps skinfold thickness, and pre-albumin
2	Sahathevan et al., 2021 [14], randomized, open-label controlled trial	Rx-29 Control-27	HD with PEW, age range 18–70 years	Rx: ONS (475 kcal, 21.7 g protein) taken 30 min after initiation of HD and at home on non-HD days combined with nutrition counseling. Control: nutrition counseling alone	6 months	Significant changes within each group: Rx: ↑ dietary energy or protein intake, ↑ mid-thigh girth, ↑ dry weight, ↑ pre-albumin Control: No change in any markers Rx group vs. control: ↑ rectus femoris thickness and CSA at midpoint, ↑ vastus intermedius thickness at midpoint; ↓ dietary energy intake, ↓ MIS, ↓ PEW; no change in appetite, handgrip strength, physical activity level, BMI, albumin, hsCRP and IL-6
3	Martin-Alemany et al., 2020 [11], randomized parallel controlled trial	Rx1-15 Rx2-15 Rx3-15	Young HD older than 18 years, mean age 29 ± SD 9.3 years	Rx1: ONS (480 kcal, 20 g protein, 56 g carbohydrate, 20 g lipid) Rx2: ONS + aerobic exercise (consisting of pedaling stationary bike during the first 2 h of the HD session) Rx3: ONS + resistance exercise (four sets of 20 repetitions for 40 min during the first 2 h of the HD sessions)	3 months	Significant changes within each group: Rx1: ↑ handgrip strength, ↑ 6MWT, ↑ TUG, ↑ body weight, ↑ BMI, ↑ albumin Rx2: ↑ handgrip strength, ↑ 6MWT, ↑ TUG, ↑ STS; ↓ albumin Rx3: ↑ handgrip strength, ↑ 6MWT, ↑ TUG, ↑ STS, ↑ weight, ↑ BMI, ↑ percentage body fat, ↑ triceps skinfold thickness, ↑ albumin Rx1 vs. Rx2 vs. Rx3: No change in mid-arm circumference, arm muscle circumference, arm muscle area, physical activity, body weight, BMI, fat mass, triceps skinfold thickness, albumin, and c-reactive protein
4	Gamboa et al., 2020 [15], prospective randomized, open-label, parallel arm trial	Rx1-6 Rx2-6	HD, older than 18 years	Rx1: ONS during HD (480 kcal, 16.7 g protein, 52.8 g carbohydrate, 22.7 g lipid) Rx2: ONS during HD (480 kcal, 16.7 g protein, 52.8 g carbohydrate, 22.7 g lipid) + resistance exercise (leg press within 30 min before each HD session)	6 months	Significant changes within each group: Rx1 and Rx2 at 3 months: ↑ markers of mitochondrial content in muscle (mtDNA copy no.); no change in mitochondrial PGC-1 alpha Rx1 and Rx2 at 6 months: ↑ forearm protein net balance, ↑ mid-thigh CSA, ↑ mid-thigh fat area; no change in mid-thigh muscle area Rx1 vs. Rx2 at 3 months: No change in forearm protein net balance, whole body protein metabolism, mtDNA copy no., mitochondrial PGC-1 alpha, mid-thigh CSA, mid-thigh muscle area, and mid-thigh fat area Rx1 vs. Rx2 at 6 months: No change in forearm protein net balance, whole body protein metabolism, mid-thigh CSA, mid-thigh muscle area, and mid-thigh fat area
5	Jeong et al., 2019 [12], randomized controlled trial	Rx1-38 Rx2-29 Control-34	HD, age range 30–80 years	Rx1: 30 g whey protein at beginning of each HD session Rx2: 30 g whey protein+ supervised exercise training (cycle ergometer up to 45 min) Control: ~150 g of a non-nutritive beverage during HD	12 months	Significant changes within each group: Rx1: at 6 months: ↑ dietary protein intake, ↑ gait speed; no change in muscle strength (leg maximal flexion force), STS, and TUG at 12 months: ↑ dietary protein intake, ↑ gait speed; no change in muscle strength (leg maximal flexion force), STS, and TUG Rx2: at 6 months: ↑ dietary protein intake, ↑ muscle strength (leg maximal flexion force), ↑ STS, ↑ TUG, ↑ gait speed at 12 months: ↑ dietary protein intake, ↑ muscle strength (leg maximal flexion force), ↑ gait speed; no change in STS and TUG Control at 6 months and at 12 months: ↑ gait speed (only at 6 months); no change in dietary protein intake, muscle strength (leg maximal flexion force), STS, and TUG Two Rx groups vs. control: No change in albumin, IL-6, and CRP
6	Ikizler et al., 2018, Aydemir et al. 2020 [16,17], randomized controlled trial	Rx1-30 Rx2-28 Rx3-27 Control-26	Moderate to severe overweight or obese CKD (stage 3 and 4), age range 18–75 years	Rx1: aerobic exercise+ calorie restriction Rx2: usual activity+ calorie restriction Rx3: aerobic exercise+ usual dietControl: usual activity+ usual diet	4 months	Significant changes within each group: Rx1: ↓ percentage body fat, ↓ body weight, ↓ F2-isoprostane, ↓ IL-6; no change in VO_2 peak_ Rx2: ↑ plasma adiponectin; ↓ percentage body fat, ↓ body weight, ↓ F2-isoprostane, ↓ IL-6; no change in VO_2 peak_ Rx3: ↓ F2-isoprostane, ↓ IL-6; no change in VO_2 peak_, percentage body fat, and body weight Rx1 vs. control: ↓ percentage body fat, ↓ body weight
7	Zilles et al., 2018 [18], prospective randomized trial	Rx1-7 HIV positive Rx2-16 HIV negative (treatment: 8, control: 8)	HD patients with and without HIV, age range 18–75 years	Rx1: ONS (250 kcal, 9.4 g protein, 25 g carbohydrates, 12.5 g lipid) consumed after HD and on non-HD days Rx2: Treatment: ONS (250 kcal, 9.4 g protein, 25 g carbohydrate, 12.5 g lipid) consumed after HD and on non-HD days Rx2: Control: no nutritional supplement	6 months	Significant changes within each group: In both Rx1 and Rx2: No change in iliopsoas muscle thickness at CSA, mid-arm circumference, subjective global assessment, BMI, subcutaneous fat thickness, albumin, c-reactive protein, IL-1beta, tumor necrosis factor alpha, and IL-6 Only in Rx1: ↑ body cell mass Significant changes in Rx1 and Rx2 Treatment combined: ↑ BMI; no change in phase angle alpha and albumin Rx1 vs. Rx2: No change in iliopsoas muscle thickness at CSA, mid-arm circumference, body cell mass, phase angle alpha, subjective global assessment, BMI, subcutaneous fat thickness, albumin, c-reactive protein, (IL)-1beta, tumor necrosis factor alpha, and IL-6
8	Martin Alemany et al., 2016 [19], randomized trial	Rx1-19 Rx2-17	HD patients older than 18 years with no physical activity (86% had a BMI <23 kg/m^2^, 83% had a serum albumin <3.8 g/dL, and 55.5% were diagnosed with PEW)	Rx1: ONS during HD (434 kcal, 19.2 g protein, 22.8 g lipid) Rx2: ONS during HD (434 kcal, 19.2 g protein, 22.8 g lipid) + resistance exercise	3 months	Significant changes within each group: In both Rx1 and Rx2: ↑ dietary energy or protein intake, ↑ mid-arm circumference, ↑ mid-arm muscle circumference, ↑ phase angle, ↑ handgrip strength, ↑ body weight, ↑ BMI, ↑ triceps skinfold thickness, ↑ percentage body fat, ↑ albumin Rx1 vs. Rx2: No change in dietary energy or protein intake, mid-arm muscle circumference, mid-arm circumference, phase angle, handgrip strength, PEW, body weight, BMI, triceps skinfold thickness, percentage body fat, and albumin
9	Hristea et al., 2016 [20], randomized controlled trial	Rx1-10 Rx2-11	Old HD patients with PEW, mean age 69.7 ± SD 14.2 years	Rx1: ONS or IDPN Rx2: ONS or IDPN+ exercise (moderate intensity cycling at the beginning of HD)	6 months	Significant changes within each group: Rx2: ↑ total energy intake, ↑ 6MWT Rx1 vs. Rx2: No change in dietary energy or protein intake, quadriceps force, lean-tissue index, PEW, BMI, fat-tissue index, albumin, pre-albumin, and c-reactive protein
10	Tomayko et al., 2015 [21], randomized controlled trial	Rx1-11 Rx2-12 Control-15	HD patients older than 30 years	Rx1: 27 g whey protein beverage (15 min before HD) Rx2: 27 g soy protein beverage (15 min before HD) Control: 2 g non caloric placebo beverage (15 min before HD)	6 months	Significant changes within each group: In both Rx1 and Rx2: ↑ gait speed, ↑ shuttle walk test; ↓ IL-6 Control: ↓ gait speed Two Rx groups vs. control: ↑ gait speed; no change in whole body lean mass, shuttle walk test, body weight, whole body fat, and albumin
11	Molsted et al., 2013 [22], randomized controlled trial	Rx1-16 Rx2-13 Control-29	Dialysis patients undergoing HD or PD, more than 18 years	Rx1: ONS (251 kcal, 9.4 g protein, 25 g carbohydrate, 12.5 g lipid) + strength training Rx2: non-protein ONS (251 kcal, 2.4 g carbohydrate, 27.3 g lipid) + strength training Control: usual care	4 months	Rx1 vs. Rx2: No change in energy intake, muscle fiber composition or size, muscle power (leg extension), muscle strength (knee extension), physical performance (chair stand test), body weight, and BMI Two Rx groups vs. control: ↑ energy intake, ↑ muscle power (leg extension), ↑ muscle strength (knee extension), ↑ physical performance (chair stand test), ↑ body weight, ↑ BMI; ↓ type 2x muscle fiber number

↑ Increase; ↓ Decrease. Rx: Treatment group; HD: hemodialysis; ONS: oral nutrition supplementation; IDPN: intradialytic parenteral nutrition; MIS: malnutrition inflammation score; BMI: body mass index; PEW: protein energy wasting syndrome; CSA: cross sectional area; hs-CRP: high sensitive c-reactive protein; IL-6: Interleukin-6; 6MWT: six-minute walk test; TUG: timed up-and-go; STS: sit-to-stand; mtDNA copy no.: mitochondrial DNA copy number; mitochondrial PGC-1 alpha: mitochondrial peroxisome proliferator-activated receptor gamma coactivator-1 alpha; VO_2 peak_: peak oxygen consumption; HIV: human immunodeficiency virus; IL-1beta: Interleukin-1 beta; PD: peritoneal dialysis.

**Table 2:** Response to exercise training in HD or PD patients.

Column	Reference, Study Design	No. of Patients	Type of Participants	Methods of Intervention	Duration	Significant Results
1	Sheshadri et al., 2020 [23,24], randomized controlled trial	Rx-30 Control-30	Undergoing HD or PD; older than 18 years	Rx: Used pedometers with counseling to increase their daily steps by 10% each week. Control: usual care	3 months intervention and 3 months post-intervention follow-up	Rx vs. control at 3 months: ↑ daily steps, ↑ heart rate variability; no change in total body muscle mass, BMI, fat mass, short physical performance battery score, and endothelial function Rx vs. control at 6 months: ↑ total body muscle mass; ↓ BMI, ↓ fat mass; no change in daily steps, short physical performance battery score, endothelial function, or heart rate variability
2	Cooke et al., 2018 [25], open-label randomized controlled trial	Rx-10 Control-10	HD; mean age 55 ± SD 16 years, mean BMI 26.4 ± SD 5.2 kg/m^2^	Rx: pedaling exercise during HD Control: usual care	4 months	Rx vs. control: ↑ waist:hip ratio; ↓ heart rate-corrected augmentation index, ↓ carotid femoral pulse wave velocity, ↓ heart rate; no change in handgrip strength, gait speed, BMI, albumin, peripheral, and central blood pressure
3	Manfredini et al., 2017 [26], randomized controlled trial	Rx-151 Control-145	HD or CAPD	Rx: low-intensity home-based personalized walking exercise Control: usual care	6 months	Rx vs. control: ↑ 6MWT; ↓ STS; no change in albumin, heart rate, and blood pressure
4	Thompson et al., 2016 [27], randomized controlled trial	Rx1-8 Rx2-7 Rx3-8 Control-8	HD; older than 18 years	Rx1: aerobic exercise (cycling) during HD Rx2: resistance exercise during HD Rx3: combined exercise (aerobic + resistance) during HD Control: routine stretching during HD	3 months	Three Rx groups vs. control: No change in 6MWT, STS, and muscle strength (one repetition maximum)
5	Bennett et al., 2016 [28], prospective stepped intradialytic resistance training randomized controlled trial	Rx1-80 Rx2-80 Rx3-68	HD; older than 18 years	Rx1, Rx2, Rx3: resistance exercise during HD. The time of commencement of the resistance training program in each HD unit was determined randomly.	Rx1: 9 months Rx2: 6 months Rx3: 3 months	With intradialytic resistance training: ↑ STS; ↓ 8-ft timed up-and-go
6	Olvera-Soto et al., 2016 [9], randomized controlled trial	Rx-30 Control-31	HD; older than 18 years, (83% with some grade of malnutrition)	Rx: resistance exercise during HD Control: usual care	3 months	Significant changes within each group: Rx: ↑ percentage body fat, ↑ arm muscle circumference, ↑ arm muscle area, ↑ handgrip strength Control: ↓ handgrip strength Rx vs. control: ↑ handgrip strength; no change in dietary protein or energy intake, percentage body fat, arm muscle circumference, and arm muscle area
7	Lewis et al., 2015 [29], randomized controlled trial	Rx1-15 Rx2-13 Rx3-17 Rx4-14 Control-22	HD; mean age 42.1 ± SD 1.5 years; healthy matched controls mean age 40.9 ± SD 2.6 years	Rx1: HD, no training Rx2: HD, endurance training Rx3: HD, strength training RX4: HD, a combination of endurance and strength training Control: normal sedentary people with no exercise training	22 weeks	Significant changes within each group: Rx2: ↑ number of capillaries in type I and IIA fibers of vastus lateralis, ↑ capillary density of vastus lateralis, ↑ capillary-to-fiber ratio Rx3: No change in the number of capillaries in any type of fibers of vastus lateralis, capillary density of vastus lateralis, and capillary-to-fiber ratio. Rx4: ↓ thickness of type I and IIA fiber CSA in vastus lateralis; ↑ capillary number in type I, IIA, and IIX fibers of vastus lateralis; ↑ capillary density of vastus lateralis, ↑ capillary-to-fiber ratio With any type of training (Rx2, Rx3, and Rx4) vs. no training (Rx1): No change in the proportion of type I, IIA, and IIX fibers in vastus lateralis muscle and succinate dehydrogenase activity in type I, IIA, and IIX fibers in vastus lateralis
8	Matsufuji et al., 2015 [30], prospective randomized parallel controlled trial	Rx-12 Control-15	HD; age range 61–79 years	Rx: chair stand exercise before HD Control: stretching exercise before HD	3 months	Rx vs. control: ↑ functional independence measure; no change in thigh circumference, knee extensor strength, 6MWT, and albumin
9	Bohm et al., 2014 [10], prospective randomized trial	Rx1-30 Rx2-30	HD; older than 18 years	Rx1: ergometer cycling during HD Rx2: pedometer home-based walking	6 months	Significant changes within each group: Rx1: ↑ STS, ↑ SR; no change in VO_2 peak_, 6MWT, and PAL Rx2: ↑ STS, ↑ SR; no change in VO_2 peak_, 6MWT, and PAL Rx1 vs. Rx2: No change in VO_2 peak_, 6MWT, STS, SR, and PAL
10	Kirkman et al., 2014 [31], randomized controlled trial	Rx1-12 Rx2-5 Control1: 11 Control2: 4	HD; older than 18 years; normal sedentary individuals	Rx1: progressive resistance training during HD Rx2: progressive resistance training in normal individuals Control1: low-intensity lower body stretching during HD Control2: low-intensity lower body stretching in normal individuals	3 months	Rx1 vs. control1: ↑ thigh muscle volume, ↑ knee extensor strength; no change in STS, 6MWT, and 8-ft get-up-and-go test Rx2 vs. control2: ↑ STS, ↑ 6MWT; no change in thigh muscle volume, knee extensor strength, and 8-ft get-up-and-go test Rx1 vs Rx2: ↑ STS (greater increase in normal group); no change in thigh muscle volume, knee extensor strength, 6MWT, and 8-ft get-up-and-go test
11	De lima et al., 2013 [32], randomized controlled trial	Rx1-11 Rx2-10 Control-11	HD; age range 18–75 years	Rx1: strength training during HD Rx2: aerobic training during HD Control: usual care	2 months	Two Rx groups vs. control: ↑ respiratory muscular strength, ↑ number of steps achieved
12	Song et al., 2012 [33], randomized controlled trial	Rx-20 Control-20	HD; older than 18 years	Rx: resistance training during HD Control: usual care	3 months	Rx vs. control: ↑ skeletal muscle mass, ↑ leg muscle strength; ↓ body fat rate; no change in visceral far area, waist circumference, arm muscle circumference, and handgrip strength
13	Chen et al., 2010 [34], randomized controlled trial	Rx-25 Control-25	HD; older than 30 years; serum albumin < 4.2 g/dL	Rx: low-intensity strength training during HD Control: stretching exercise	6 months (48 exercise sessions)	Rx vs. control: ↑ lean whole-body mass, ↑ leg lean mass, ↑ knee extensor strength, ↑ short physical performance battery score, ↑ self-reported physical function, ↑ leisure-time physical activity; ↓ whole-body fat mass
14	Kopple et al., 2007 [35], randomized controlled trial	Rx1-10 Rx2-15 Rx3-12 Control1-14 non-exercising HD Control2-20 normal people	HD; age range 25–65 years; healthy matched controls	Rx1: endurance training during HD Rx2: strength training before HD Rx3: endurance + strength training before and during HD Control1: usual care HD Control2: non-exercising normal	22 weeks	Significant changes within each group: Rx1: ↑ muscle mRNA in IGF-1 and IGFBP-2 Rx2: ↑ muscle mRNA in IGF-IEa and IGF-1 Rx3: In muscle, ↑ IGF-IEa mRNA, ↑ IGF-IEc mRNA, ↑ IGF-II mRNA Significant changes in Rx1, Rx2, and Rx3 combined: ↑ vastus lateralis muscle mRNA in IGF-IEa, IGF-IEc, IGF-IR, IGF-II, IGFBP-2, and IGFBP-3, ↑ muscle IGF-1, ↑ anthropometry derived fat-free mass; ↓ muscle mRNA of myostatin, ↓ skinfold thickness, ↓ anthropometry-derived percentage body fat and total body fat; no change in dietary protein or energy intake, c-reactive protein, tumor necrosis factor alpha, and IL-6
15	Cheema et al., 2007 [36], randomized controlled trial	Rx-24 Control-25	HD; older than 18 years	Rx: high-intensity resistance training during HD Control: usual care	3 months	Rx vs. control: ↓ thigh muscle lipid infiltration, ↓ c-reactive protein; ↑ total strength, ↑ mid-arm circumference, ↑ mid-thigh circumference, ↑ body weight, ↑ BMI; no change in dietary energy or protein intake, total or subcutaneous fat of the mid-thigh, muscle CSA, 6MWT, and habitual physical activity
16	Storer et al., 2005 [37] †	Rx-12 HD Control1-12 non-exercising MHD Control2-12 normal people	HD; Normal matched controls	Rx: stationary-cycle endurance training during HD Control1: non-exercising HD patients Control2: non-exercising normal	9 weeks	Significant changes within each group: Rx: ↑ quadriceps strength, ↑ leg-press fatigability, ↑ stair-climb time, ↑ 10 m walk, ↑ timed up-and-go, ↑ VO_2 peak_, ↑ endurance time Non-exercise HD and normal subjects: no changes.
17	Vilsteren et al., 2005 [38], randomized controlled trial	Rx-53 Control-43	HD; older than 18 years	Rx: strength training (before HD) plus cycling (during HD) Control: usual care	3 months	Rx vs. control: ↑ reaction time, ↑ lower extremity muscle strength; no change in VO_2 peak_

↑ Increase; ↓ Decrease; Rx: Treatment group; HD: hemodialysis; PD: peritoneal dialysis; SD: standard deviation; CAPD: continuous ambulatory peritoneal dialysis; BMI: body mass index; CSA: cross sectional area; IL-6: Interleukin-6; 6MWT: six-minute walk test; STS: sit-to-stand; SR test: sit-and-reach test (measurement of flexibility); PAL: physical activity level; VO_2 peak_: peak oxygen consumption; IGF: insulin-like growth factor; IGF-IR: insulin-like growth factor–insulin receptor; IGFBP: insulin-like growth factor binding protein. ^†^ This was a non-randomized controlled trial.
